# Mechanisms underlying the effects of alpine wetland vegetation succession on the community assembly and functional potential of *nirK*-type denitrifier

**DOI:** 10.3389/fmicb.2026.1914409

**Published:** 2026-09-07

**Authors:** Jiao Tang, Shijia Zhou, Ni Zhang, Chen Chen, Kelong Chen

**Affiliations:** 1 Qinghai Province Key Laboratory of Physical Geography and Environmental Process, College of Geographical Science, Qinghai Normal University, Xining, China; 2 Key Laboratory of Tibetan Plateau Land Surface Processes and Ecological Conservation (Ministry of Education), Qinghai Normal University, Xining, China

**Keywords:** alpine wetlands, community assembly, insurance effect, NirK-type denitrifiers, vegetation succession

## Abstract

Denitrification in alpine wetlands critically regulates regional nitrogen budgets and N_2_O emissions, and *nirK*-harboring denitrifiers represent an important functional group involved in nitrite reduction during denitrification. Spatial heterogeneity across distinct plant communities can modulate soil microenvironments, thereby shaping N_2_O production, consumption, and emission dynamics. Here, using a space-for-time substitution approach, we investigated four successional stages—early (BS), transitional (CR), middle (EN), and mature (LS)—in the alpine wetlands of Qinghai Lake. High-throughput sequencing combined with a null model framework was applied to characterize the coupling between microbial community patterns and their functional potential. Vegetation succession stimulated microhabitat divergence characterized by shifts in total nitrogen (TN), total carbon (TC), and the carbon-to-nitrogen ratio (CNR). This environmental filtering induced a directional microbial succession, culminating in the highest diversity at the LS stage. The CNR emerged as the primary driver accounting for community variance, mediating a microhabitat niche separation that selectively enriched key heterotrophic denitrifiers (such as *Sinorhizobium* and *Paracoccus*). The βNTI null model revealed that deterministic processes governed community assembly; notably, the underlying assembly rules underwent a fundamental shift as succession advanced, becoming dominated by heterogeneous selection at the LS stage. Co-occurrence networks peaked in complexity at the EN stage but reorganized into a more consolidated, modular, and highly competitive topology at the LS stage. Furthermore, functional profiling indicated that while the BS stage was functionally oriented toward nitrate respiration and nitrogen fixation, the LS stage exhibited a distinct advantage in possessing predicted denitrification-related functional profiles. In summary, vegetation succession reshaped *nirK*-type denitrifier communities via environmental filtering, steering the assembly mechanisms from stochasticity toward determinism; meanwhile, the “insurance effect” of the diverse community structure sustained stable functional output amid nutrient fluctuations. This study clarifies that vegetation succession is a core driving force of the nitrite-reducing potential of *nirK*-type denitrifiers, providing micro-scale mechanistic insights for the restoration of degraded alpine wetlands, vegetation reconstruction, and the maintenance of regional nitrogen homeostasis under changing climates.

## 1 Introduction

Nitrous oxide (N_2_O), a potent non-CO_2_ greenhouse gas, possesses a global warming potential approximately 273 times that of carbon dioxide (CO_2_); furthermore, its stratospheric decomposition yields nitric oxide, which catalytically destroys the ozone layer (Champeecharoensuk et al., 2023). Alpine wetland ecosystems retain vast soil nitrogen pools due to suppressed microbial decomposition under chronic low temperatures, rendering them significant potential sources of N_2_O emissions (Yang et al., 2021). Within these vulnerable terrestrial ecosystems, denitrification—the reduction of ${\text{NO}}_{3}^{-}$ to N_2_O and N_2_–represents the core pathway regulating gaseous nitrogen loss and mediating net nitrogen removal (Zumft, 1997). Driven by watershed hydrological shifts alongside climate warming and anthropogenic disturbances, the wetland habitats of the Qinghai-Tibet Plateau, a vital ecological barrier, are undergoing intense vegetation succession (Tian et al., 2023). This process substantially restructures belowground microbial communities and their co-occurrence networks (Wu B. et al., 2023). Currently, conflicting paradigms persist regarding how vegetation succession drives denitrifying communities and subsequent gaseous nitrogen emissions. Ample evidence indicates that advancing succession markedly escalates plant biomass, particularly belowground root biomass, which continuously inputs organic carbon and available nitrogen into the soil via root exudates and litter, thereby alleviating nutrient limitations (Choudhury et al., 2022). Dominated by these “resource-acquisition” plants, a strong positive selection and enrichment effect is exerted on denitrifying functional groups (Ma L. et al., 2020), typically causing incomplete denitrification potential and N_2_O emission fluxes to increase significantly along the successional gradient (Kastl et al., 2014). However, an alternative body of empirical evidence suggests that increases in plant diversity can intensely exacerbate exploitative competition for inorganic nitrogen at the rhizosphere interface between plants and microorganisms (Saghaï et al., 2022). Highly efficient plant nitrogen uptake may severely deplete the soil nitrate pool, thereby suppressing the abundance and expression of denitrifying populations and ultimately limiting N_2_O release (Ameer et al., 2025). Furthermore, specific secondary metabolites secreted by mid-to-late successional plants can inhibit the activity of particular nitrogen-transforming taxa within the rhizosphere (Legay et al., 2014). Consequently, a profound disentanglement of the mechanisms driving denitrifying microbes across different successional stages is of paramount importance for predicting future gaseous nitrogen emission patterns in alpine wetlands.

Soil denitrification is an exceptionally intricate biological process catalyzed by a cascade of stepwise reducing enzymes. Among these, nitrite reduction (${\text{NO}}_{2}^{-}\to$NO) constitutes the rate-limiting step of the entire denitrifying pathway, directly dictating the structural differentiation of denitrifying communities (Braker et al., 1998). This pivotal step is catalyzed by either the copper-containing nitrite reductase (encoded by the *nirK* gene) or the cytochrome cd1-containing nitrite reductase (encoded by the *nirS* gene) (Heylen et al., 2006), both of which exhibit profound phylogenetic diversity and distinct ecological niches (Bremer et al., 2007; Cantera and Stein, 2007). Beyond these twin core nitrite-reducing genes, the complete denitrification process concurrently relies on nitrate-reducing genes (e.g., *narG*) that initiate the pathway, as well as nitrous oxide reductase genes (e.g., *nosZ*) responsible for the final conversion of N_2_O to N_2_ (Kandeler et al., 2006; Yoshida et al., 2009). The linkages between vegetation succession and denitrification are inextricably intertwined. Plant community turnovers, by modifying belowground microenvironments (such as fine root proportions, root tissue density, and root C/N ratios), offer a diverse array of microniches for heterogeneous selection, thereby altering the assembly mechanisms of denitrifying communities (Yuan et al., 2021). Concurrently, these shifts directly govern the physical equilibrium between “producers” and “reducers” along the denitrification pathway, fundamentally modulating overall denitrification efficiency (Kanaan, 2025). Substantial empirical progress has been achieved regarding *nirK*-type denitrifying communities across primary successional transects in the Qinghai-Tibet Plateau and analogous alpine-polar regions. For instance, along a century-scale chronosequence formed by glacial retreat in the Austrian Alps, the *nirK* gene exhibited prominent “pioneer colonizer” characteristics during early successional stages (Deiglmayr et al., 2006; Tscherko et al., 2005). Similarly, along a primary successional sequence in a Dutch coastal salt marsh, *nirK*-type denitrifying communities demonstrated remarkable structural variability and stress-tolerant adaptation during early phases of environmental perturbation (Salles et al., 2017). Furthermore, multi-factor manipulative experiments on the Qinghai-Tibet Plateau revealed that the topological network structure of *nirK* communities was tightly coupled with the accumulation of recalcitrant alkyl carbon (Yuan et al., 2020). In permafrost gradient studies, denitrification-related genes displayed a conspicuous vertical enrichment pattern in deeper soil horizons (Kang et al., 2024). Moreover, within arid saline-alkali habitats, parasitic plants (such as *Cistanche tubulosa*) have been shown to modulate the structural potential of nitrogen-cycling functional groups by restructuring the metabolic profiling of the host rhizosphere bacterial community (Tang et al., 2025).

As an endorheic alpine lake located in the northeastern Qinghai-Tibet Plateau, the Qinghai Lake basin encompasses alpine wetlands that are currently experiencing intense vegetation succession driven by climate warming and water level fluctuations (Hou et al., 2023; Sun et al., 2025). Advancing vegetation succession directly alters regional soil hydrodynamic properties (e.g., soil moisture, saturated hydraulic conductivity, and porosity) alongside available nutrient inputs. Elucidating the impacts of vegetation succession on *nirK*-type denitrifying microorganisms is of paramount importance; this process not only prone to generating substantial amounts of the potent greenhouse gas N_2_O, but also serves as a critical ecological barrier intercepting and purifying land-derived nitrate loss in alpine oligotrophic soils, thereby preventing water eutrophication (Song et al., 2019; Zhao et al., 2022). However, a critical knowledge gap remains regarding how the restructuring of plant biomass and the concurrent modification of habitat microenvironments induced by such succession select for specific belowground functional guilds. To address this, we employed an ecological “space-for-time (SFT) substitution” approach (Pickett, 1989; Walker et al., 2010) to investigate four representative successional stages around the Qinghai Lake wetlands: early succession (*Blysmus sinocompressus* community; BS), transitional succession (*Carex regescens* community; CR), middle succession (*Elymus nutans* community; EN), and mature succession (*Leymus secalinus* community; LS). By coupling high-throughput sequencing with bioinformatics tools such as null model analysis, this study aims to clarify the impacts of vegetation successional sequences on the structural turnover and community assembly of *nirK*-type denitrifiers, while exploring how their nitrogen removal functions are maintained via the “insurance effect” under environmental drivers. This research will deepen our micro-scale understanding of nitrogen removal mechanisms in alpine endorheic wetlands, providing a foundational scientific basis for ecological restoration and nitrogen homeostasis in these fragile regions. Specifically, we address the following two questions: (1) How do the spatial characteristics and taxonomic composition of *nirK*-type denitrifying communities evolve along the vegetation successional gradient in the Qinghai Lake wetlands? (2) What is the core environmental drivers steering the evolution of these communities, and how do their assembly mechanisms and denitrification potentials exhibit an “insurance effect” in response to succession?

## 2 Materials and methods

### 2.1 Overview of the study area

**Figure 1:**
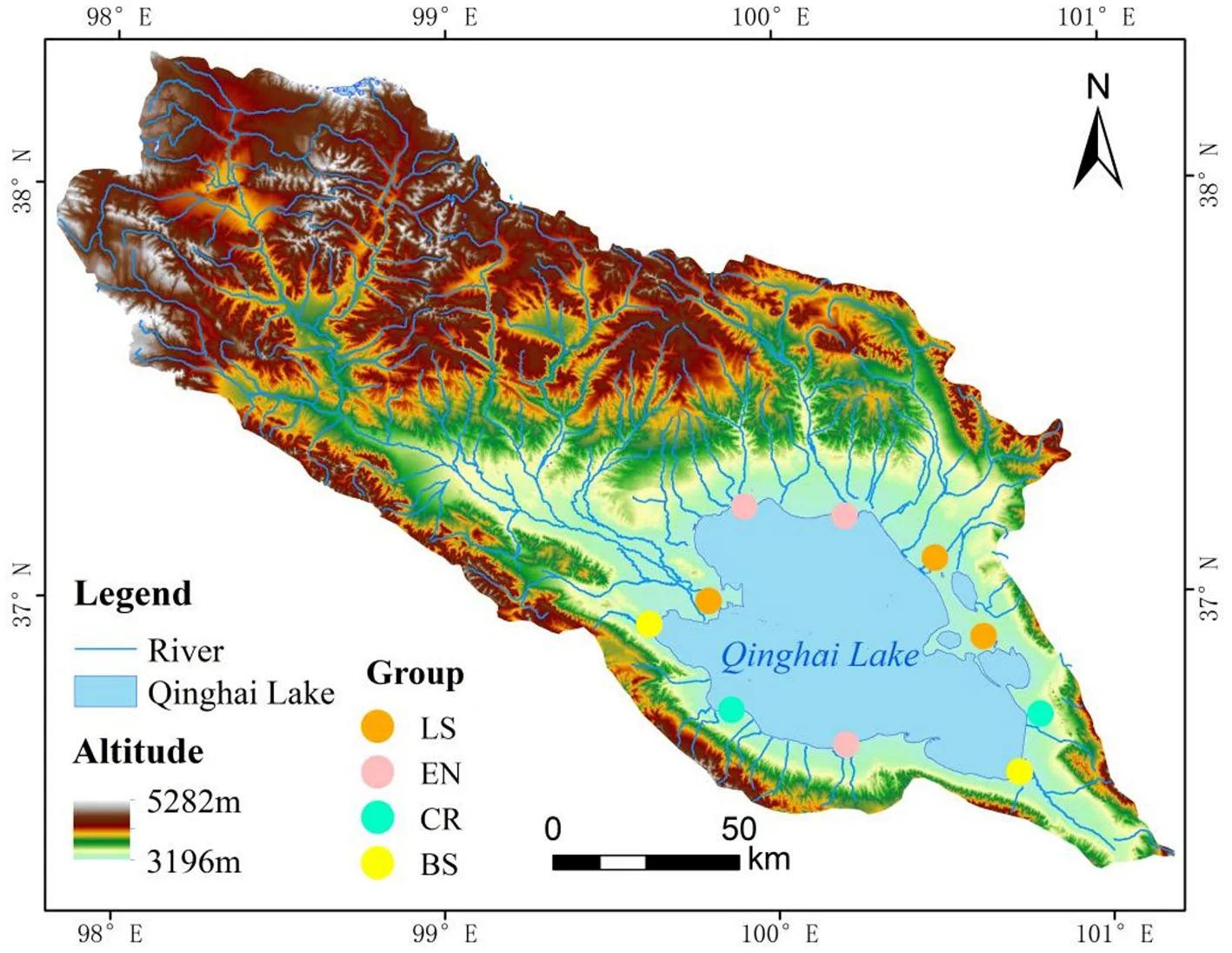
Overview of the study area and sampling sites.

The study area is located in the Qinghai Lake basin (36°15′-38°20′ N, 97°50′-101°20′ E), northeastern Qinghai Province, China (Figure 1). This region lies at the intersection of three major bioclimatic zones: the arid northwest, the alpine southwest, and the monsoonal east. The basin has a distinct semi-arid plateau climate with pronounced annual temperature fluctuations, with extremes ranging from −31 °C to 28 °C. Mean annual precipitation varies between 2.91 m and 6.05 m, and the mean annual temperature ranges from −1.0 °C to 4.0 °C. The terrain is highly undulating, with elevations spanning from approximately 3,007 m at the lowest point to 5,285 m at the peak; the water surface of Qinghai Lake itself lies at an elevation of roughly 3,194 m. Alpine meadow soils represent the dominant soil type, interspersed with sandy loam soils in certain areas. The vegetation composition is diverse, primarily comprising steppe, alpine meadow, shrubland, alpine sparse vegetation, desert, and wetlands (Ma et al., 2026; Wu et al., 2022; Wu X. et al., 2023).

### 2.2 Plot design and sampling

The space-for-time substitution approach was used to infer potential successional patterns rather than to directly reconstruct a confirmed temporal sequence. The selected sites shared comparable regional climatic, geomorphological, hydrological, and soil backgrounds, and the dominant plant communities represented widely recognized vegetation stages in the Qinghai Lake wetland succession sequence. Following chronosequence criteria, 10 typical sites with similar environmental backgrounds were selected across the basin, comprising two early successional plots (BS), two transitional plots (CR), three middle successional plots (EN), and three mature successional plots (LS). This multi-site design captured regional spatial heterogeneity while minimizing confounding environmental noise and strictly avoiding ecological pseudo replication, ensuring that vegetation succession remained the sole dominant variable driving environmental differentiation among plots. Field sampling was conducted in August 2020 during the peak growing season to capture the strongest ecological function signals. Within each site, five independent herbaceous quadrats (spaced > 5 m apart) were randomly established as biological replicates, generating a total of 50 independent soil and biomass samples (BS = 10, CR = 10, EN = 15, and LS = 15) for subsequent physicochemical analyses and high-throughput sequencing. For sample collection, aboveground biomass (AB) was obtained by clipping all vegetation at the soil surface within a representative one-quarter area of each quadrat, removing debris, and sealing the samples. Belowground biomass (BB) and soil samples were collected using a multi-point soil coring method combined with a sieving and washing procedure. Specifically, five topsoil cores (0–10 cm depth) were taken along an S-shaped transect within each quadrat and thoroughly mixed into one composite sample. Roots were then separated, and only physiologically active live roots—identified by color, elasticity, and toughness—were retained as BB, with all dead roots rigorously discarded.

### 2.3 Determination of soil physicochemical properties

Field-fresh soil samples were cleared of visible plant debris, passed through a 2 mm sieve, and randomly divided into two subsamples. Subsamples designated for *nirK* gene sequencing were immediately flash-frozen in liquid nitrogen, while the remaining portions for chemical analyses were stored at 4 °C. Soil NH^4+^-N was extracted with 2 M KCl and determined by continuous flow analysis (FUTURA, France). Total carbon (TC) and total nitrogen (TN) were quantified by high-temperature combustion using an elemental analyzer (Vario EL III, Germany). Soil pH was measured *in situ* in a 1:2.5 (w/v) soil-to-water suspension using a pH meter (Mettler Toledo, Switzerland). Soil temperature and moisture at each plot were recorded *in situ* at multiple points using a handheld soil thermometer and a moisture sensor, respectively. All aboveground (AB) and belowground (BB) biomass samples were oven-dried at 60 °C to constant weight and then weighed.

### 2.4 DNA extraction and *nirK* gene sequencing

Total genomic DNA was isolated from 0.5 g of fresh soil using the PowerSoil DNA Isolation Kit (MoBio Laboratories, USA) according to the manufacturer's instructions. The quality and structural integrity of the extracted DNA templates were assessed via 2% agarose gel electrophoresis (operated at 80 V for 40 min), and the DNA concentrations were uniformly normalized to 1 ng μL^−1^. For the *nirK* functional gene, targeted PCR amplification was executed using the primer copper583F (5′-TCATGGTGCTGCCGCGKGACGG-3′) and copper909R (5′-GAACTTGCCGGTKGCCCAGAC-3′). Thermal cycling was performed in an ABI GeneAmp 9700 thermal cycler (Applied Biosystems, USA) under the following conditions: an initial denaturation at 95 °C for 5 min; followed by 30 standard cycles (95°C 30 s, 55°C 30 s, 72°C 45 s); with a final extension step at 72 °C for 10 min. High-throughput sequencing of the purified amplicons was subsequently conducted on the Illumina NovaSeq 6000 platform.

### 2.5 Statistical analysis

By assembling (PANDASEQ) and quality-filtering (PRINSEQ) the obtained raw data, low-quality sequences and potential chimeras were removed to secure a high-quality dataset for downstream analyses. Operational taxonomic units (OTUs) were clustered at a 97% identity threshold using the UCLUST algorithm, laying the foundation for investigating denitrifying microbial composition. Microbial richness and evenness across sampling sites were evaluated using multiple alpha diversity metrics, including the Shannon index and Chao1 estimator. Mean differences among treatment groups were determined via Wilcoxon rank-sum tests; additionally, analysis of similarities (ANOSIM) was employed to verify the heterogeneity of community structures among groups. All data processing, statistical computing, and rarefaction curve plotting were performed in R (v4.0.5), primarily utilizing bioinformatics packages such as Microbiota Process (Callahan et al., 2016).

Microbial functional variations among different sample groups were excavated via linear discriminant analysis effect size (LEfSe), with the LDA score threshold set at 2.0 to screen statistically significant biomarkers and identify signature taxa. To elucidate the functional potential and taxonomic identities associated with the *nirK*-harboring microbial community, representative sequences (or their translated amino acid sequences) of all identified *nirK* OTUs were aligned against the NCBI non-redundant (nr) protein database using BLASTX to retrieve top-hit matches and determine unambiguous taxonomic classifications at the genus level (Altschul et al., 1990; Sayers et al., 2022). Subsequently, a host-taxon-to-function mapping framework was implemented: the metabolic functional capabilities inherent to these identified *nirK*-carrying host genera were annotated by cross-referencing established microbial functional databases (Douglas et al., 2020). The annotated functional attributes were then integrated with the *nirK* gene relative abundance matrix to quantify and visualize the predicted metabolic profiles of the *nirK*-carrying taxa across different successional stages. Regarding environmental driving mechanisms, detrended correspondence analysis (DCA) clarified data response models, thereby determining the deployment of linear-model-based redundancy analysis (RDA) to quantify the explanatory contributions of environmental physicochemical factors to the spatial distribution of the denitrifying community. Relationships between dominant genera and environmental factors were calculated using Spearman rank correlation coefficients and visualized via correlation heatmaps, with association strengths validated by significance tests (*P* < 0.05). The geographical distribution map of sampling sites was constructed using ArcGIS (v10.8).

To infer the ecological processes governing *nirK*-harboring denitrifier community assembly, the β-nearest taxon index (βNTI) was calculated based on β-mean nearest taxon distance (β MNTD) and null distributions generated by randomizations. Pairwise comparisons were categorized according to standard ecological thresholds: |βNTI|>2 indicated deterministic selection (βNTI >+2 for variable selection and βNTI <−2 for homogeneous selection). For pairwise comparisons falling within the neutral zone (|βNTI| ≤ 2), Raup-Crick Bray-Curtis (*RC*_*bray*_) metrics were further calculated to partition stochastic processes, distinguishing dispersal limitation (*RC*_*bray*_>+0.95), homogenizing dispersal (*RC*_*bray*_ <−0.95), and undominated drift (|*RC*_*bray*_| ≤ 0.95). Concurrently, co-occurrence networks were constructed separately for each successional stage using OTUs retained after abundance and prevalence filtering. Pairwise Spearman rank correlation coefficients were calculated, and only robust, statistically significant correlations were retained for network construction. Key topological properties—including node and edge numbers, average degree, modularity, edge sign proportions, and clustering coefficients—were quantified using the igraph package in R and visualized in Gephi.

## 3 Results

### 3.1 Variations in soil physicochemical properties and plant biomass along the vegetation successional gradient

**Figure 2:**
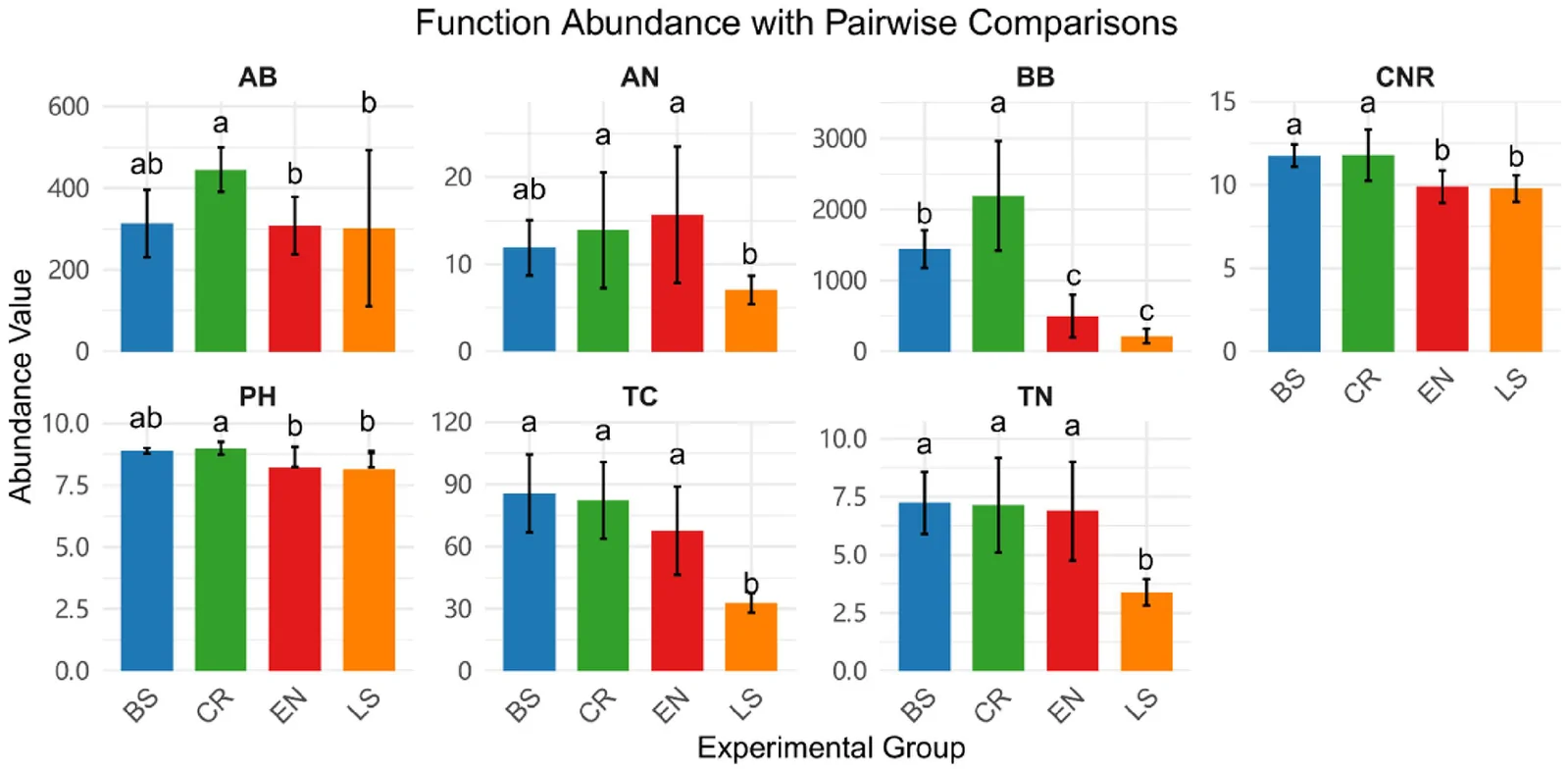
Soil physicochemical properties and plant biomass characteristics across different vegetation successional stages in the Qinghai Lake wetlands. Differences between groups labeled with the same letter above the bar chart are not significant (*p* > 0.05), while differences between groups labeled with different letters are significant (*p* < 0.05). AB, aboveground biomass; BB, belowground biomass; TC, total carbon; TN, total nitrogen; CNR, carbon-to-nitrogen ratio; AN, ammonium nitrogen; pH, soil pH. BS, early succession; CR, transitional succession; EN, middle succession; LS, mature succession.

Analysis of soil physicochemical properties and biomass characteristics revealed that the vegetation successional process exerted significant effects on key environmental variables (Figure 2). Specifically, as succession advanced toward the LS stage, soil carbon and nitrogen nutrients, along with plant biomass, exhibited pronounced stage-specific differentiation. BB peaked significantly at the CR stage (*P* < 0.05) and subsequently decreased markedly at the EN and LS stages (*P* < 0.05). Concurrently, soil TC and TN contents remained at relatively high levels across the BS, CR, and EN stages without significant differences (*P* > 0.05), but showed a sharp and significant decline at the LS stage (*P* < 0.05). Furthermore, soil CNR was significantly higher at the BS and CR stages than at the EN and LS stages, while the ammonium nitrogen (AN) content decreased to its significantly lowest value at the LS stage (*P* < 0.05). Variation in soil pH primarily manifested as a distinct separation between the early (BS, CR) and late (EN, LS) successional phases. Along the successional gradient, soil pH decreased significantly, with values at the EN and LS stages being lower than those at the CR stage (*P* < 0.05), indicating a noticeable soil acidification trend in the wetland microhabitats at the LS stage.

### 3.2 Sequencing depth quality control, alpha diversity, and taxonomic intersection profiling of *nirK*-type denitrifying communities

**Figure 3:**
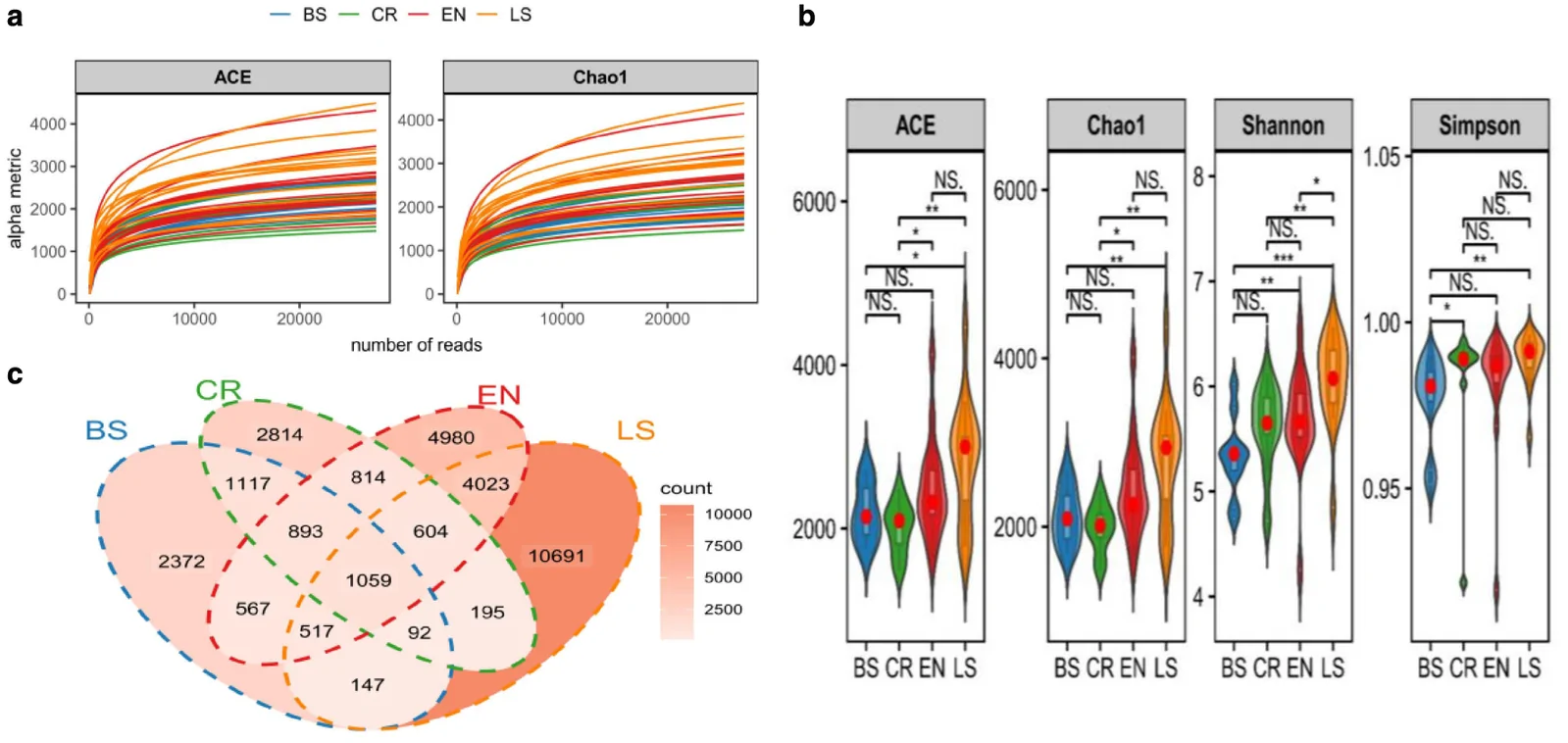
Comprehensive profile of *nirK*-type denitrifying microbial diversity across different vegetation successional stages in the Qinghai Lake wetlands. **(a)** Rarefaction curves of soil samples; **(b)** Alpha diversity indices of the *nirK*-type denitrifying communities; **(c)** Venn diagram illustrating the shared and unique OTUs. ^*^p < 0.05; ^**^p < 0.01; ^***^p < 0.001; NS: *p* > 0.05. BS, early succession; CR, transitional succession; EN, middle succession; LS, mature succession.

When the sequencing depth reached approximately 28,000 reads, the rarefaction curves for all samples plateaued markedly (Figure 3a). This indicates that the current sequencing depth has extremely high sample representativeness, sufficient to capture the vast majority of *nirK*-type denitrifying microbial species. Alpha diversity analysis revealed that the vegetation successional process exerted significant shaping effects on the *nirK*-type denitrifying community diversity (Figure 3b). Specifically, the ACE and Chao1 indices, which reflect species richness, remained relatively stable with no significant differences among the BS, CR, and EN stages (*p* > 0.05). However, as the wetland succession advanced to the LS stage, both richness metrics exhibited a sharp and significant increase, becoming significantly higher than those at the BS and CR stages *(p* < 0.05, *p* < 0.01). Echoing the richness dynamics, the Shannon index, which reflects community diversity and evenness, demonstrated that the LS stage was significantly higher than the BS (*p* < 0.001), CR (*p* < 0.01), and EN stages (*p* < 0.05). Furthermore, the Simpson index remained low at the BS stage, increased significantly upon transitioning to the CR stage (*p* < 0.05), and maintained highly significant levels at the LS stage (*p* < 0.01). These results indicate that advancing vegetation succession substantially elevates the species richness and overall diversity structure of the *nirK*-type denitrifying community.

Venn diagram analysis revealed that 1,059 shared OTUs were detected across the four successional stages (Figure 3c). Along the successional trajectory, the number of unique OTUs within each stage exhibited distinct dynamic changes. The number of unique OTUs was relatively low at the BS stage, whereas it expanded dramatically to 10,691 unique OTUs upon reaching the LS stage. This trend demonstrates that as the plant community stabilizes and habitat heterogeneity increases, the mature stage harbors a broader suite of highly specialized microbial taxa.

### 3.3 Beta diversity spatial differentiation and multi-level taxonomic responses of *nirK*-type denitrifying communities

**Figure 4:**
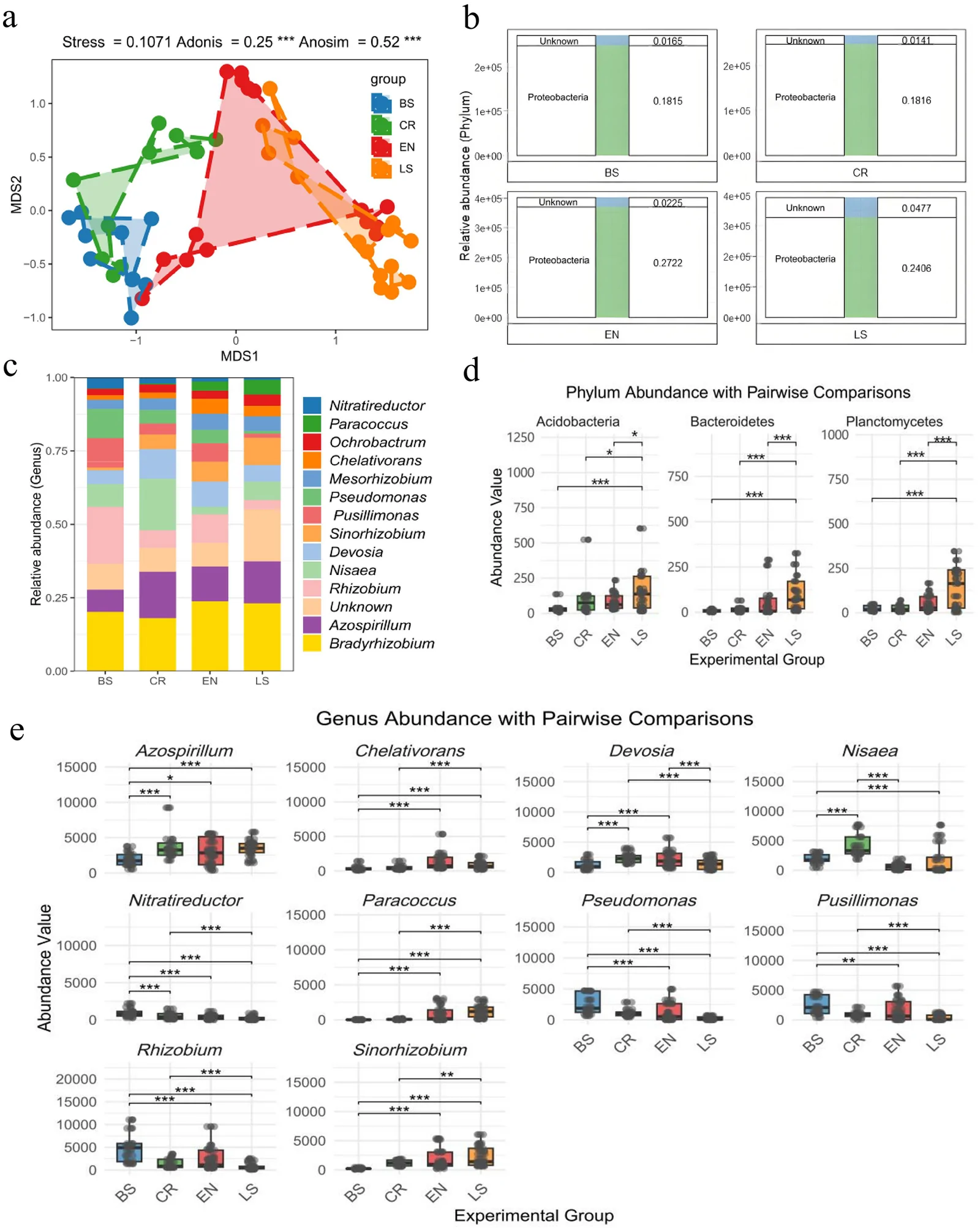
Community taxonomic profiles and composition responses of *nirK*-type denitrifiers across different vegetation successional stages in the Qinghai Lake wetlands. **(a)** Non-metric multidimensional scaling (NMDS) analysis based on the nirK-type denitrifying community structures. **(b)** Relative abundance of *nirK*-type denitrifying bacteria at the phylum level; **(c)** Significance tests for differences in the abundances of major phyla; **(d)** Relative abundance of *nirK*-type denitrifying bacteria at the genus level; **(e)** Significance analysis for differences in the abundances of core denitrifying genera. ^*^p < 0.05; ^**^p < 0.01; ^***^p < 0.001. BS: early succession; CR, transitional succession; EN, middle succession; LS, mature succession.

Non-metric multidimensional scaling (NMDS) confirmed that *nirK*-type denitrifier communities differed significantly among successional stages (*R*^2^ = 0.25, *p* < 0.001; *R* = 0.52, *p*<*0.001*; Figure 4a). The BS and CR stage overlapped partially and displayed a tight spatial arrangement, indicating high community homogeneity. In contrast, the EN and LS stages occupied larger convex hulls, showing elevated intragroup heterogeneity and a clear, stepwise successional turnover along the ordination gradient.

At the phylum level, *Proteobacteria* dominated all successional stages, reaching a maximum relative abundance of 27.22%, whereas unclassified microorganisms (Unknown) showed an increasing trend with succession (Figure 4b). Significance testing on major phyla revealed that *Acidobacteria, Bacteroidetes*, and *Planctomycetes* exhibited coherent responses (Figure 4c). Their abundances increased significantly along the successional gradient and peaked at the LS stage (*p* < 0.05, *p* < 0.001), indicating their growing prominence in denitrification as the wetland matures.

At the genus level, *Bradyrhizobium* was dominant across all stages (Figure 4d). However, core genera underwent pronounced shifts with succession: *Sinorhizobium* and *Mesorhizobium*, classic heterotrophic denitrifiers, increased markedly at the LS stage, while Rhizobium and Pseudomonas, which were abundant at the CR stage, declined sharply at the LS stage. Analysis of core *nirK*-type denitrifying genera further revealed distinct successional dynamics (Figure 4e). *Sinorhizobium* and *Paracoccus* showed extremely low abundances at the BS and CR stage but became highly enriched at the LS stage (*p* < 0.01). In contrast, *Nitratireductor* decreased stepwise (*p* < 0.01), and *Pseudomonas, Pusillimonas*, and *Rhizobium* maintained high abundances at BS and CR but dropped significantly at the LS stage (*p* < 0.001). *Devosia* and *Nisaea* peaked at the CR stage and then declined markedly. This drastic restructuring of core genera at the mature stage underscores the selective pressure exerted by habitat evolution in shaping the target denitrifying communities.

### 3.4 Co-occurrence networks and topological properties of the *nirK*-type denitrifying community

**Figure 5:**
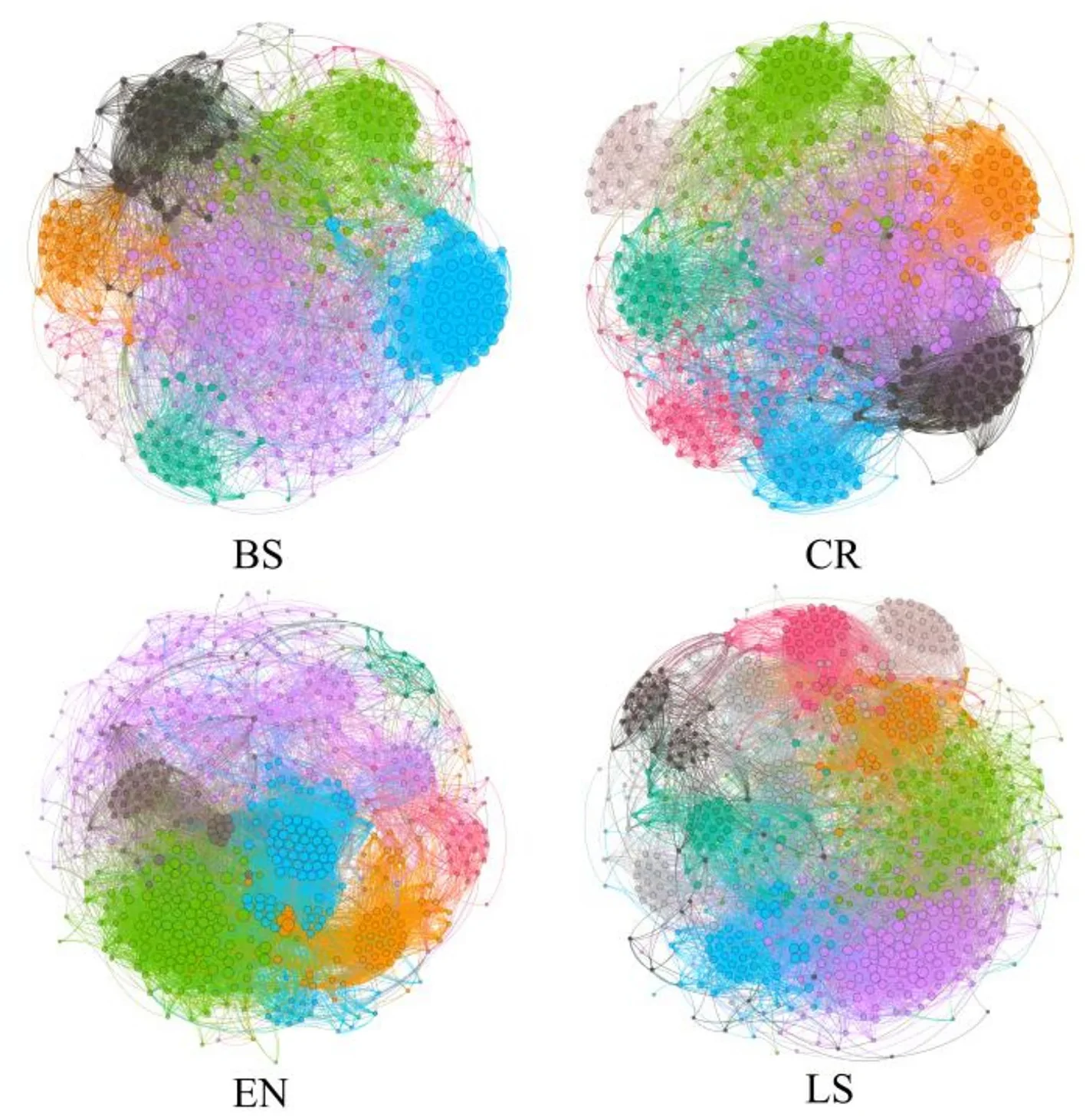
Molecular ecological network analysis of the *nirK*-type denitrifying microbial communities across different successional stages in the Qinghai Lake wetlands. Nodes represent distinct operational taxonomic units (OTUs); edges connecting the nodes represent statistically significant correlations between species (*p* < 0.05). Nodes are color-coded based on their modular attributes, with nodes sharing the same color belonging to the same ecological module. BS, early succession; CR, transitional succession; EN, middle succession; LS, mature succession.

The co-occurrence networks exhibited pronounced spatiotemporal variations and structural reorganization across different successional stages (Figure 5, Supplementary Table S1). During the BS, CR, and EN stages, the *nirK*-type denitrifying communities displayed dense connectivity and a high average degree. Notably, the network size and total number of edges peaked at the EN stage, reflecting the most complex topological configuration. Conversely, as succession advanced to the LS stage, network complexity contracted markedly, with both the total number of edges and the average degree declining. However, the number of nodes and the modularity index concurrently reached their maximum values across the entire chronosequence. This topological transition manifested as a clearer differentiation of the modular architecture, with the community tending to cluster into specific ecological modules. Furthermore, positive correlations overwhelmingly dominated the network edges across all successional stages. Negative edges, representing non-cooperative interactions, dropped to their lowest level at the CR stage, followed by a continuous stepwise increase through the EN and LS stages, ultimately reaching the highest value at the LS stage.

### 3.5 Evolution of putative functional profiles and their “insurance effects” in response to succession

**Figure 6:**
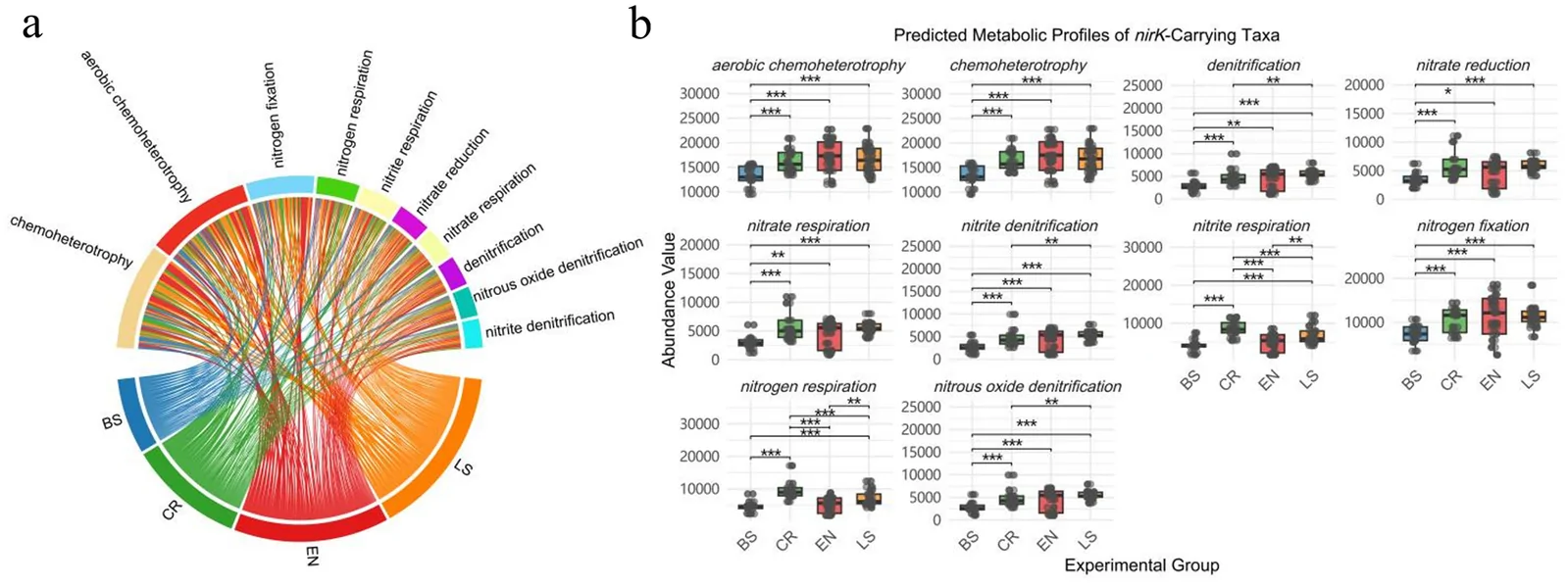
Structural and functional differentiation characteristics of the *nirK*-type denitrifying microbial communities. **(a)** Chord diagram analysis showing the metabolic functional potentials of the *nirK*-type denitrifying microbial communities across different successional stages in the Qinghai Lake wetlands. **(b)** Inferred metabolic functional profiles of *nirK*-carrying microbial taxa. ^*^p < 0.05; ^**^p < 0.01; ^***^p < 0.001. BS, early succession; CR, transitional succession; EN, middle succession; LS, mature succession.

Inferred functional profiling of the *nirK*-carrying microbial taxa further elucidated the specific impacts of vegetation succession on the ecological functional potentials of the denitrifying communities. The chord diagram analysis revealed that the predicted core heterotrophic metabolic functions, such as aerobic chemoheterotrophy and chemoheterotrophy, exhibited a distinct redistribution across different successional stages (Figure 6a). Notably, the predicted nitrogen fixation function displayed a relatively higher inferred relative abundance during the CR stage. Furthermore, the functional categories associated with nitrogen metabolism exhibited notable successional shifts in their inferred relative abundances. During the CR stage, the predicted functional potentials for nitrate respiration, nitrate reduction, and nitrite respiration were relatively enhanced; conversely, during the LS stage, the inferred functional potentials for denitrification-related steps, nitrite denitrification, and nitrous oxide denitrification increased markedly, reaching their highest inferred abundance levels at this mature stage.

Inter-group comparisons of the abundances of these denitrification functions further quantitatively validated the aforementioned differentiation patterns (Figure 6b). The statistical analysis of inter-group significance demonstrated that chemoheterotrophy, aerobic chemoheterotrophy, and nitrogen fixation all exhibited significant increases upon entering the CR and EN stages (*p* < 0.001), and remained significantly elevated at the LS stage. In contrast, nitrate respiration, nitrate reduction, and nitrite respiration peaked at the CR stage, followed by significant declines in both the EN and LS stages (*p* < 0.05, *p* < 0.001). However, the core denitrification pathways—including total denitrification, nitrite denitrification, and nitrous oxide denitrification—displayed a continuous stepwise increasing dynamic. Their abundances at the LS stage were significantly higher than those at the BS stage (*p* < 0.01), which is highly consistent with the phased successional shifts observed in the structure and abundance of the *nirK*-type denitrifying community in this study.

### 3.6 Core environmental drivers, contribution estimates, and community assembly mechanisms

**Figure 7:**
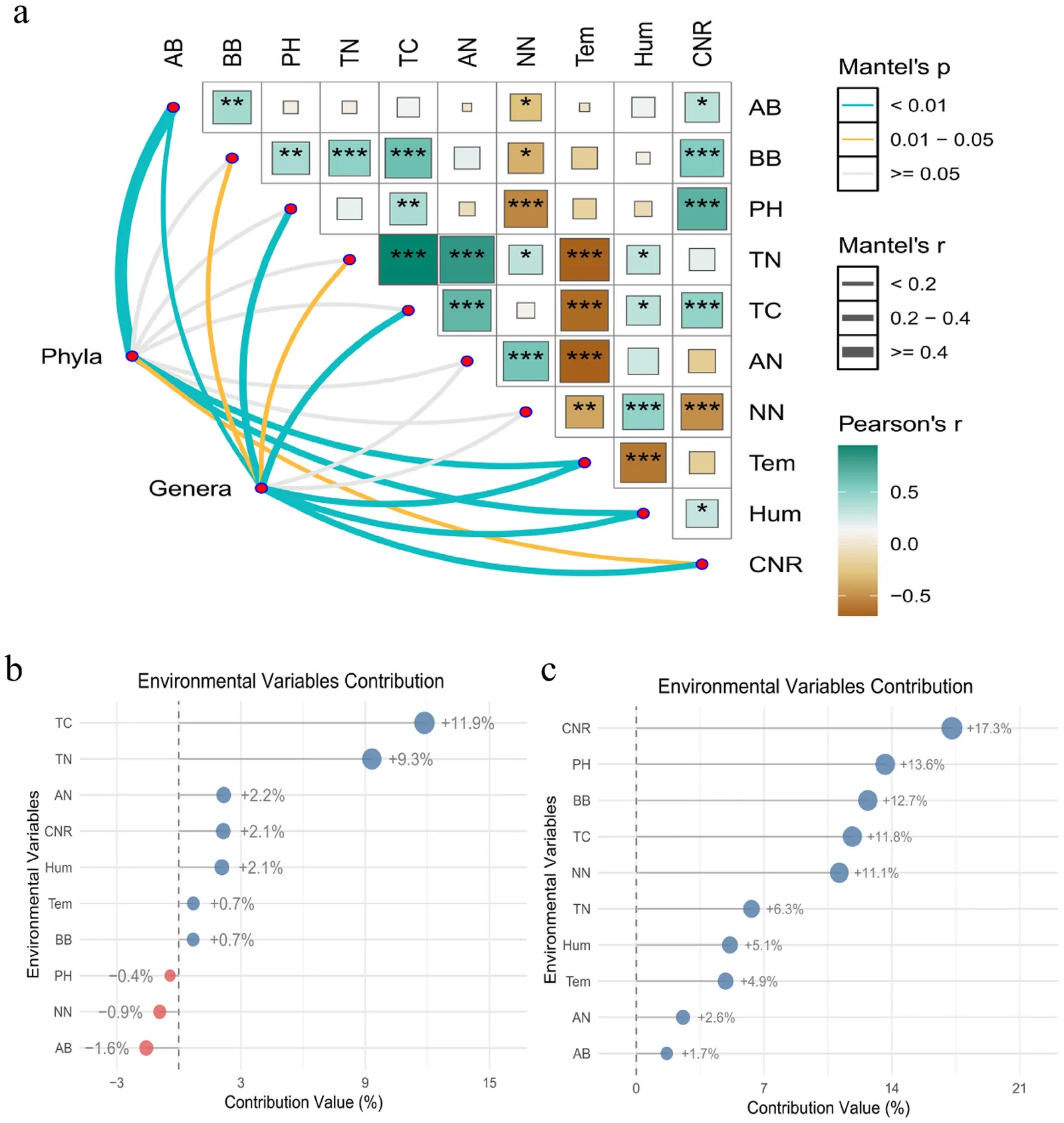
Correlation and driver contribution analysis between environmental variables and the *nirK*-type denitrifying microbial communities. **(a)** Mantel test analysis evaluating the correlations between environmental factors and the *nirK*-type denitrifying microbial community structures in the Qinghai Lake wetlands. **(b)** Contribution rate prediction analysis identifying the critical environmental drivers for the alpha diversity of the *nirK*-type denitrifying microbial communities in the Qinghai Lake wetlands. **(c)** Contribution rate prediction analysis identifying the critical environmental drivers for the community structure and functional potentials of the *nirK*-type denitrifying microbial communities in the Qinghai Lake wetlands. ^*^p < 0.05; ^**^p < 0.01; ^***^p < 0.001.

Mantel test and correlation matrix results demonstrated that plant biomass and soil carbon-nitrogen nutrients are the most critical positive environmental factors regulating the reorganization of the *nirK*-type denitrifying community in the Qinghai Lake wetlands (Figure 7a). Specifically, total carbon (TC) showed significant positive correlations with belowground biomass (BB), total nitrogen (TN), ammonium nitrogen (AN), and the carbon-to-nitrogen ratio (CNR) (*p* < 0.001). Concurrently, TN also displayed significant positive correlations with AN, TC, and BB (*p* < 0.001). Within the resource-enrichment matrix, CNR was significantly and positively correlated with BB, pH, and TC (*p* < 0.001). In contrast, environmental temperature (Tem) showed negative correlations with most other factors, with significant negative correlations observed between Tem and TN, TC, AN, and soil humus (Hum) (*p* < 0.001). Phylum- and genus-level analyses revealed that the phylum-level community was significantly regulated by aboveground biomass (AB), Hum, and CNR (*p* < 0.05, *p* < 0.01). The genus-level community was insensitive to variations in AN and nitrate nitrogen (NN) (*p* > 0.05) but showed significant positive correlations with all other environmental drivers (*p* < 0.05, *p* < 0.01). Overall, the genus-level community demonstrated greater sensitivity to environmental fluctuations.

Further predictions of the contributions of environmental variables to microbial community diversity and structure revealed that the explanatory power of individual factors exhibited pronounced indicator-specific variations. For community diversity (Figure 7b), TC (+11.9%) and TN (+9.3%) were the two most critical positive drivers, whereas AN (+2.2%) and CNR (+2.1%) showed moderate positive explanatory capacities. Notably, AB contributed the largest negative effect (−1.6%), while BB had a relatively minor contribution (+0.7%). Regarding the mechanisms underlying community structure and functional potentials, the explanatory power of each factor underwent a comprehensive reorganization (Figure 7c). CNR (+17.3%) and pH (+13.6%) were the two most influential positive drivers of structural differentiation, closely followed by BB (+12.7%), TC (+11.8%), and NN (+11.1%). In contrast, the explanatory share of AB (+1.7%) dropped to its lowest level, demonstrating a limited role in driving structural variation.

**Figure 8:**
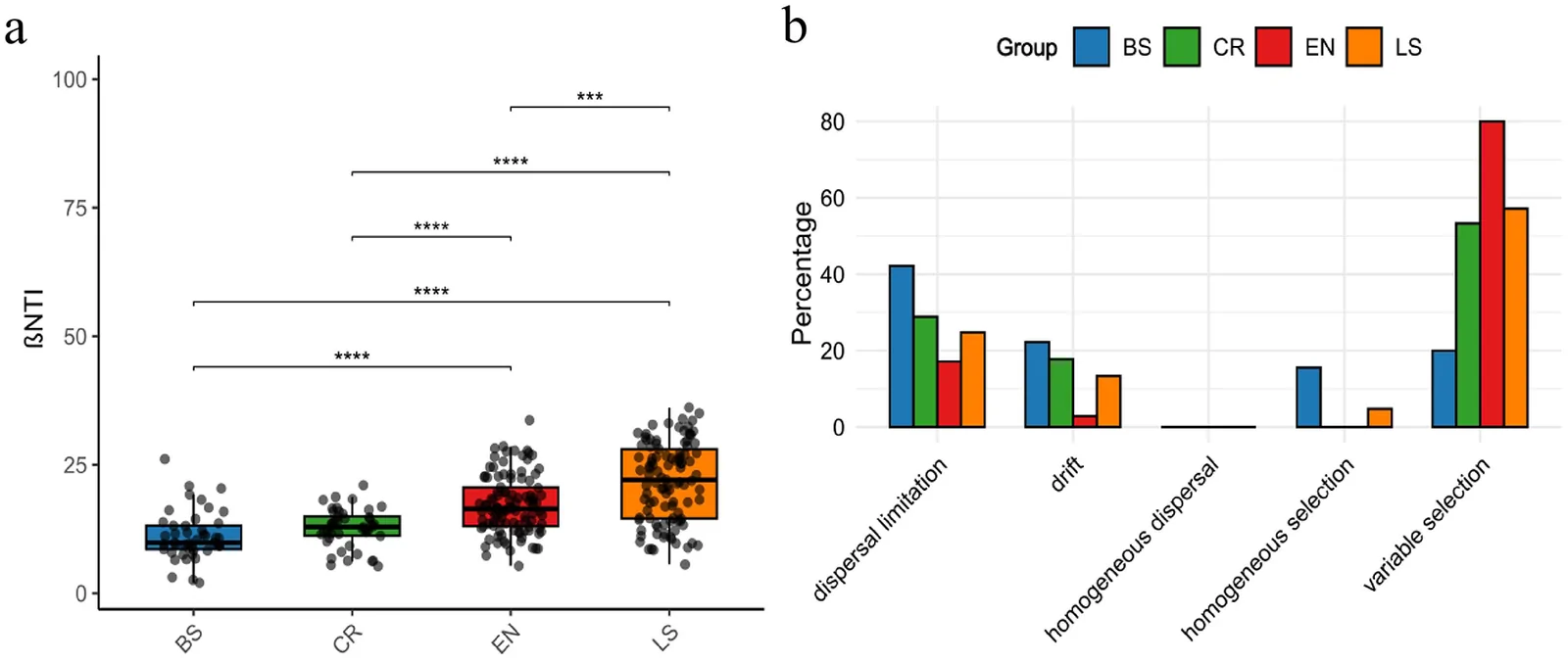
Community assembly dynamics and relative process contributions across different successional stages. **(a)** βNTI analysis evaluating the community assembly mechanisms of the *nirK*-type denitrifying microbial communities across different successional stages in the Qinghai Lake wetlands. ^***^p < 0.001; ^****^p < 0.0001. **(b)** Partitioning of the relative contributions of deterministic and stochastic processes driving the assembly of the *nirK*-type denitrifying microbial communities across different successional stages in the Qinghai Lake wetlands. BS, early successional stage; CR, transitional stage; EN, middle successional stage; LS, mature successional stage.

To elucidate the ecological processes governing microbial community composition, βNTI analysis was performed (Figure 8a). Across the four vegetation successional stages, βNTI values exhibited a continuous stepwise and statistically significant increasing trend with succession. Inter-group comparisons confirmed that, except for the non-significant difference between the BS and CR stages, βNTI values at the EN and LS stages were significantly higher than those at the preceding two stages (*p* < 0.001). This indicated that the ecological selection pressure driving denitrifier community turnover was directionally enhanced as the wetland habitat evolved. Further quantitative partitioning of community assembly processes revealed a clear successional dynamic shift between deterministic and stochastic processes in regulating the reorganization of the *nirK*-harboring denitrifier community (Figure 8b). At the BS stage, stochastic processes exhibited a relatively higher contribution to community assembly. However, as succession advanced, particularly at the LS stage, the assembly mechanism underwent a marked transition, with variable selection within deterministic processes emerging as a primary driving force, while the relative contribution of stochastic processes decreased substantially. In sharp contrast, homogeneous selection, also belonging to deterministic processes, was extremely low at this stage. This strong asymmetric contrast demonstrates that the wetland habitat underwent directional resource differentiation during the mature successional stage, constituting the fundamental ecological driver shaping the structure and functional potential of the *nirK*-type denitrifying community in the Qinghai Lake wetlands.

## 4 Discussion

### 4.1 Subsurface microhabitat remodeling driven by vegetation succession and the taxonomic response of *nirK*-type denitrifying communities

Over long-term succession, shifts in aboveground plant communities disrupt the existing belowground equilibrium, driving the adaptive reset of specific nitrogen-cycling microbial taxonomic configurations (Wang et al., 2021). Our results showed that at the LS stage, despite significant reductions in TC, TN, and AN due to phased resource depletion, the alpha diversity indices of the *nirK*-type community peaked against this trend, accompanied by the enrichment of up to 10,691 unique OTUs. A study on wetland sediments in the Florida Everglades reached contrasting conclusions, suggesting that the abundance of heterotrophic denitrifying communities harboring the *nirK* gene was significantly and positively correlated with total carbon, extractable organic carbon concentrations, and potential denitrification rates; consequently, their community size and activity contracted markedly in unfertilized, resource-poor oligotrophic sites (Kim et al., 2016). Conversely, a study on plant succession in the Yellow River Delta coastal wetlands of China reached similar conclusions, showing that as the system advanced from bare land to later successional stages, plant colonization-induced niche differentiation generally enhanced soil microbial species richness and community heterogeneity (Gao et al., 2021). Although the adaptive increase in microbial alpha diversity across stages in that typical coastal chronosequence primarily manifested as a long-term cumulative trend without statistically significant differences, the peak in species carrying capacity and evenness at the final stage highlighted the positive role of plant community stability in shaping belowground microbial configurations. In comparison, the “resource drawdown coupled with diversity peaking” pattern observed in our study exhibited a distinct non-linear decoupling. This peak achieved at a “nutrient trough” indicates that community assembly in alpine wetlands freed itself from absolute reliance on parent material nutrient concentrations, reflecting a unique adaptive buffering resilience (Jing et al., 2026). The patchy remodeling of subsurface microhabitats by the *Leymus* community at the mature stage effectively buffered resource limitations through “niche expansion” (Bais et al., 2006; Guyonnet et al., 2017), thereby perfectly demonstrating the ecological “insurance effect” through the adaptive reorganization of fine-scale taxonomic configurations (Griffiths and Philippot, 2013; Yachi and Loreau, 1999).

Further molecular taxonomic insights into this reorganization revealed that *Proteobacteria* and *Bradyrhizobium* were the dominant phylum and genus, respectively, in the alpine wetland soil community. This finding is consistent with results from the Ebinur Lake inland saline wetland in China, which suggested that because the wetland substrate is subjected to long-term high-intensity hydrological dynamics or localized salinization constraints, *Proteobacteria* and *Bradyrhizobium* serve as core *nirK*-type functional groups in both the host rhizosphere and bulk soil (Niu et al., 2022). Another large-scale altitudinal gradient study on the Qinghai-Tibet Plateau further substantiated this conclusion. Focusing on broader geographic and environmental filtering pressures, that study confirmed that along drastic habitat variations induced by the vertical alpine gradient, variable selection within belowground deterministic processes dominated; in particular, localized soil pH acted as an overriding filter, directly governing the spatial stability and evolution of functional generalists such as *Bradyrhizobium* at large spatial scales (Kou et al., 2021). However, in contrast to the traditional model of single-factor linear sorting, we observed a distinct “convective” phenomenon: the dominant genera at the BS stage, *Nitratireductor* and *Pseudomonas*, decreased significantly by the LS stage, whereas *Sinorhizobium* and *Paracoccus* became specifically enriched at the LS stage. The underlying mechanism lies in the BB expansion and intense CNR fluctuations triggered at the LS stage, which elicited deep root foraging of substrates and clonal exclusion. Together with the *in situ* verified localized soil acidification, these three micro-environmental filters collectively contracted the niche breadths of generalists. Ultimately, by directionally recruiting specific taxa equipped with specialized denitrification and stress-resistance strategies, a functional and structural reset was successfully achieved under resource-limited conditions in the alpine wetland.

### 4.2 Topological reorganization and niche differentiation mechanisms of *nirK*-type denitrifying co-occurrence networks along the successional chronosequence

As vegetation progresses toward the mature stage, localized microhabitat acidification and nutrient depletion act as environmental filters, forcing the belowground functional community to reorganize its internal topological connections to counteract external resource stress (Chen et al., 2026; Fierer and Jackson, 2006). Analysis of microbial network topological parameters revealed that the co-occurrence network of the *nirK*-type denitrifying community exhibited a topological transition characterized by initial expansion at the EN stage followed by contraction at the LS stage, reflecting an active adaptive strategy of this functional group under extreme resource limitation in the alpine wetland. Network complexity peaked at the EN stage and subsequently declined significantly at the LS stage. This pattern is highly consistent with the observed phenomenon that increasing habitat stress induces a decline in network complexity along an alpine altitudinal gradient (Chen et al., 2022). Furthermore, a comparative study of alpine meadows and alpine steppes in northern Tibet confirmed similar topological differentiation, indicating that long-term hydrothermal constraints and nutrient deficiency strongly drive the structural contraction of community networks to reduce energy consumption and maintain system stability (Chen et al., 2025). However, the topological contraction observed at the LS stage in this study is fundamentally distinct from network collapse under intense anthropogenic disturbance. A study on agricultural intensification demonstrated that under disturbance, the rapid loss of keystone taxa triggers irreversible “degenerative disassembly” of microbial networks, characterized by chaotic collapse of connectivity and fragmentation of modular structures (Banerjee et al., 2019). In contrast, the decline in network connectivity at the LS stage in our study represents a highly ordered “active structural response” under the dual environmental filters of moisture and nutrients. Rather than simply destroying the random interactions within the functional community, it pruned inefficient, energy-intensive peripheral links, triggering a more advanced mechanism of “modular consolidation,” thereby averting the internal structural collapse similar to that observed under intensive anthropogenic disturbance.

Notably, although network connectivity declined at the LS stage, the modularity index increased significantly, indicating that the community completed a fundamental structural transformation from “competitive expansion” at the BS stage to “functional specialization” at the LS stage. The highly modular architecture observed at the LS stage is strongly supported by a meta-analysis of co-occurrence networks based on over 20,000 global samples from the Earth Microbiome Project (EMP) (Ma B. et al., 2020). That study emphasized that the refined restructuring of network modules is a direct visual imprint of niche filtering and habitat specialization on community topological configuration, with high modularity reflecting the precise positioning of microorganisms within specific micro-niches. This topological differentiation driven by habitat specialization was profoundly validated dynamically in a study on the developmental stages of calcareous glacier forelands in the Alps (Mandolini et al., 2025). That study pointed out that deterministic selection and environmental filtering imposed by local soil substrates constitute the core driving force shaping belowground microbial network topologies toward high community clustering and efficient cooperative modules. The topological reorganization of the *nirK* network observed at the LS stage in the alpine wetland is not a passive degradation caused by nutrient impoverishment; instead, the community refined its external interactions to intensely concentrate limited niche energy within tightly connected internal functional modules. This transition toward high modularity successfully achieved the adaptive persistence and stabilization of the functional community in the extreme alpine mature habitat, laying the physical and structural foundation for the counter-trend peak of denitrification pathways.

### 4.3 Successional turnover of specific nitrite reduction pathways and functional confirmation of the “insurance effect”

The underlying ecological purpose of the adaptive resetting of microbial taxonomic composition and the refined modular specialization of co-occurrence networks is to maximize the potential of core ecosystem processes under adverse conditions by reconfiguring specific functional gene pools (Berlemont et al., 2014; Siles et al., 2021). Functional potential analysis indicates that the metabolic potential of the functional microbiome exhibits precise successional stage turnover. During the BS and CR stages, the system primarily performed baseline nitrate respiration and nitrogen fixation. Upon transitioning into the LS stage, however, the predicted denitrification-related functional profiles—specifically nitrite denitrification and nitrous oxide denitrification—denitrification, nitrite denitrification, and nitrous oxide denitrification—all exhibited significant counter-trend peaks. This pathway turnover highly mirrors findings from high-latitude forest-wetland ecotones, which suggested that specific predicted functional pathways involved in belowground nitrogen cycling, particularly those of the denitrifying community, do not passively degenerate along habitat gradients; instead, driven by plant types and traits, they release strong, stage-specific metabolic potentials during different developmental phases (Wu D. et al., 2023). This logic of active plant regulation and functional pathway recruitment is further supported by research in the Qinling Mountains ecosystem (Lyu et al., 2024). That study demonstrated that native dominant vegetation undergoing long-term *in situ* development can significantly elevate the relative abundance and metabolic potential of core nitrogen cycling pathways, such as denitrification and nitrite denitrification, through its specific rhizosphere habitat filtering. This functional enrichment phenomenon mediated by wetlands or mature native vegetation has also been confirmed *in situ* in a study of natural wild rice wetland systems (McMillian, 2021). In contrast, a study along the alpine steppe successional sequence in the Qilian Mountains on the eastern margin of the Qinghai-Tibet Plateau reached the opposite conclusion (Zhao et al., 2022). They argued that as succession advances toward the late mature stage, specific belowground functional pathways regulating core element cycling, such as ectomycorrhizal fungal pathways, do not experience a counter-trend peak. Instead, they undergo interruption and a precipitous, linear decline, accompanied by progressive soil acidification and the phased depletion of carbon, nitrogen, and phosphorus resources, ultimately shrinking to the lowest point of the entire sequence at the final successional stage. Similarly, a research pointed out that in highly disturbed, intensively managed agricultural habitats (such as maize rotation croplands) that are structurally simplified and lack the buffering capacity of long-term natural succession, denitrification and related nitrogen metabolic pathways exhibit devastating functional down-regulation when facing substrate deficits, failing to maintain efficient metabolic output (Liang et al., 2020).

The marked divergence between our findings and these conventional models may be attributed to the robust “insurance effect” exerted at the system level by the high alpha-diversity taxonomic configurations accumulated during the long-term natural successional maturation of the enclosed Qinghai Lake wetland system. More importantly, even though TN and AN fell to their lowest levels across the entire sequence at the LS stage, the functional potential of the key nitrite reduction step (associated with denitrification) remained firmly in an absolutely dominant position within the functional profile. This distinct mismatch between functional performance and substrate nutrient depletion indicates that the maintenance of community functioning is not simply constrained by resource supply. Approaching successional maturity, the denitrifying community streamlined redundant interspecific connections, directionally enriched diverse specialized functional genera such as *Sinorhizobium* and *Paracoccus*, and drove the metabolic architecture toward high efficiency and specialization. During this process, although cosmopolitan generalists (e.g., *Pseudomonas*) declined in activity due to resource tightening, the specialized taxa rapidly initiated alternative nitrogen-removal pathways within the “nutrient vacuum window,” successfully achieving immediate functional compensation. Concurrently, environmental filtering prompted a contraction of the network topology, whereas the relative abundance of specific nitrogen metabolic pathways rose against the trend. The synergistic enhancement between these two processes ultimately ensured that the system maintained stable nitrite reduction efficiency (a key step in denitrification) and nitrogen removal efficacy even under conditions of extreme substrate deprivation.

### 4.4 The transition from stochasticity to “variable selection” in community assembly

Over the long-term chronosequence of alpine wetland succession, the shift in the ecological niche occupancy and assembly balance of belowground functional communities is inherently governed by the dominant role of “variable selection” (Daniel et al., 2019; Stegen et al., 2012). Results based on the βNTI null model framework demonstrated that during the BS stage, because the soil habitat at the initial developmental phase possessed strong resource buffering capacity, the selection pressure from environmental filtering had not yet crossed the threshold; consequently, functional community assembly was overall dominated by stochastic processes. However, as vegetation succession advanced toward the LS stage, the sharp expansion of belowground biomass triggered by the colonization of the dominant plant (*Leymus secalinus*), along with significant *in situ* soil acidification and the progressive, deep depletion of available nutrients from the parent material, caused a migration of local microhabitat selection pressures. This completely shifted the assembly mechanisms, which became absolutely dominated by “variable selection” within deterministic processes. This transformation in community assembly dynamics, catalyzed by spatial development and environmental constraints, highly mirrors the conclusions drawn from a long-term successional chronosequence of permafrost thaw (Doherty et al., 2020). Utilizing the βNTI model, that study confirmed that with long-term natural development and the intensification of local hydrologic and physicochemical stressors, the assembly mechanism of belowground bacterial communities undergoes a classic, fundamental leap “from stochastic to deterministic.” Conversely, this assembly trajectory exhibits an opposite evolutionary direction compared to many systems subjected to intense external disturbances and drastic substrate fluctuations. For instance, research exploring functional community assembly under different land-use and exogenous disturbance modes indicated that although high-intensity inputs of conventional inorganic chemical fertilizers similarly induced severe soil acidification, the artificial injection of fertilizers provided high concentrations of buffering substrates; this directly caused the assembly mechanism of functional communities responsible for specific nitrogen cycling (AOB) to degenerate into a purely stochastic process (Ma et al., 2023). Similarly, related studies have shown that habitat homogenization and available nutrient fluctuations brought about by alien plant invasions instead substantially enhanced the stochastic ecological drift of bacterial communities, causing the contribution rate of stochastic processes to rise against the trend (Chen and Wen, 2021). This divergence can likely be attributed to the lack of exogenous nutrient inputs during the long-term natural successional maturation of the alpine closed wetland in Qinghai Lake. When substrates plunged to their lowest trough due to stage-specific consumption during the LS stage, the microscopic survival squeeze imposed by local nutrient bottlenecks and acidification constituted an impassable “deterministic environmental filtering barrier,” thereby eliminating intolerant, peripheral stochastic generalists.

Notably, despite severe resource limitations during the LS stage, the functional communities directionally screened out via “variable selection” exhibited a strong assembly resilience that engaged in non-linear selective resonance with environmental filtering during spatial turnover. This phenomenon has been indirectly validated by a long-term study on contaminated mining soils (Wang et al., 2025). That study confirmed that under severe local and extreme chemical adversity, the disordered dispersal of belowground microorganisms is blocked, and intense “deterministic environmental filtering” drives systemic restructuring, thereby specifically enriching a fraction of highly stress-resistant, functionally specialized dominant taxa. This dynamic mechanism of adaptively reshaping interaction patterns under stress filtering also aligns with the core ecological thesis established in long-cycle biofilm community succession experiments (Brislawn et al., 2019). That paper explicitly stated that to cope with the mature phase characterized by progressive resource limitation and physical space compression, communities will actively “forfeit the priority effect” and widespread random dispersal of early stages, turning instead to deterministic turnover and taxonomic restructuring to maintain the functional robustness of the mature system. In the LS stage of alpine wetland succession around Qinghai Lake, intense “variable selection” blocked the disordered stochastic drift characteristic of the early and middle stages, systematically eliminating early-to-mid-stage baseline generalists (such as *Pseudomonas*), while directionally recruiting and absolutely enriching diverse specialized core nitrogen-removal genera. This evolution of community aggregation catalyzed by strong deterministic variable selection mechanistically explains—at the level of community assembly—the previously mentioned taxonomic configuration resetting, the adaptive counter-trend peak of diversity, and the highly modular functional specialization. Ultimately, it explains why the full-pathway denitrification potential could still deliver an absolutely dominant output even when parent material nutrients were at the lowest trough of the entire sequence, providing the underlying biological support for nitrogen control and ecological stability in alpine wetlands.

### 4.5 Limitations of the current study and future perspectives

Although this study provides a systematic analysis of the *nirK*-harboring denitrifier community along a natural successional trajectory in an alpine wetland—ranging from taxonomic configurations and co-occurrence network interactions to functional pathway turnovers and β NTI null model assembly mechanisms—several critical limitations remain to be addressed.

First, the lack of synchronized subsurface *N*_2_*O* gas flux measurements hinder the establishment of a direct, quantitative link between microbial community reorganizations, the counter-trend enrichment of specific pathways, and actual greenhouse gas emission dynamics. Although statistical analyses of the relative abundances of potential nitrogen-cycling functional groups indicated that core complete denitrification pathways—including the inferred total denitrification, nitrite denitrification, and nitrous oxide denitrification—achieved stepwise and significant counter-trend peaks at the LS stage, these taxonomically inferred functional potentials cannot be definitively validated at the ecosystem process or gas release levels without supporting *in situ* gas flux data.

Second, the actual potential denitrification rates (PDR) of the soil were not directly quantified. As a pivotal kinetic indicator of gaseous nitrogen loss and core ecological barrier homeostasis in enclosed alpine wetlands, the omission of rate measurements restricts our ability to directly infer true functional activities and nitrogen removal efficiencies from community topological reconstructions, such as the structural transition from “competitive expansion” to “functional division of labor” at the LS stage. Consequently, current conclusions rely heavily on compositional abundance profiles, network consolidation attributes, and metabolic potential allocations, leaving it unverified whether the belowground nutrient troughs and localized acidification effects remodeled by vegetation at successional maturity physically altered the absolute denitrification rates per unit soil volume. Additionally, regarding soil nitrogen indicators, this study measured only ammonium nitrogen and total nitrogen, omitting nitrate nitrogen. Although nitrate nitrogen is more active in wetland nitrogen cycling and more sensitive to environmental shifts, the lack of foresight regarding nitrogen turnover dynamics during the initial experimental design prevented us from comprehensively evaluating the immediate regulatory effects of nitrate supply on the denitrifying community, representing another limitation in our environmental parameter configuration. Moreover, the synchronous quantification of *nirS*-type denitrifiers was not performed in this study. We recommend that future investigations integrate the *nirK* and *nirS* lineages, as well as the *nosZ* gene, to construct a more comprehensive landscape of the denitrifying community, thereby complementing the current compositional findings with functional gene-level insights.

Third, while the classical “space-for-time substitution” approach employed in this study effectively captures long-term successional dynamics, it inherently entails certain limitations. This method tends to overlook fine-scale spatial heterogeneities induced by local microtopography in alpine wetlands, as well as short-term pulse-like disturbances exerted on variable selection processes by seasonal hydrothermal dynamics. Furthermore, it must be noted that the successional sequence constructed in this study represents a scientific inference under the space-for-time framework rather than an absolute temporal sequence derived from direct, long-term monitoring of permanent plots. Although the current division of successional stages aligns macroscopically with the vegetation succession patterns of the Qinghai Lake wetlands, additional long-term monitoring data from permanent plots are required to rigorously validate the absolute reliability of this inferred trajectory. Therefore, future research will prioritize long-term *in situ* monitoring of this successional sequence to further confirm its generalizability.

## 5 Conclusions

Vegetation succession was closely associated with the community assembly and predicted functional potential of *nirK*-harboring denitrifiers in alpine wetlands. As succession progresses toward the LS stage, microbial alpha diversity indices and unique OTU richness peak against the environmental trend, indicating that niche expansion induced by plant colonization during the mature stage significantly enhances the species carrying capacity of the system. In terms of hierarchical taxonomic composition, the denitrifying community maintains a system-wide absolute dominance of *Proteobacteria* and *Bradyrhizobium*. However, the successional process strongly drives strategic turnovers at lower taxonomic echelons, manifested as the directional recruitment and enrichment of *Sinorhizobium* and *Paracoccus* at the LS stage, whereas genera such as *Nitratireductor, Pseudomonas*, and *Rhizobium* decline sharply under intense habitat filtering. Concurrently with the taxonomic configuration turnover, the complexity and connectivity of the community co-occurrence network peak at the EN stage, subsequently undergoing a convergent reorganization toward highly modular specialization and a strongly competitive architecture at the LS stage. Functional predictions further reveal a phased turnover of metabolic potentials; the system primarily executes baseline nitrate respiration and nitrogen fixation during the early-to-mid successional stages, whereas it displays substantial functional compensation advantages across the core predicted denitrification-related functional profiles—nitrate respiration, nitrite denitrification, and nitrous oxide denitrification—upon entering the LS stage. Multivariate driver predictions and null model analyses ultimately substantiate that the alpha diversity of this functional community is positively driven by TC and TN availability, while its community turnover and metabolic functions are synergistically regulated by CNR, localized pH (acidification trends), and BB. With the advancement of alpine wetland succession, the assembly mechanism of belowground functional microorganisms shifts completely from stochastic drift dominance to deterministic “variable selection” control, endowing the system with a unique adaptive buffering resilience at nutrient troughs through precise functional niche positioning.

## Data Availability

The datasets presented in this study can be found in online repositories. The names of the repository/repositories and accession number(s) can be found in the article/Supplementary Material.
