# Supplementary material for: Mechanisms underlying the effects of alpine wetland vegetation succession on the community assembly and functional potential of nirK-type denitrifier
Source: Front Microbiol. 2026 Sep 7;17:1914409. doi: 10.3389/fmicb.2026.1914409 (PMC13590569; doi:10.3389/fmicb.2026.1914409)
Supplement: Supplementary file 1 [file Table_1.DOCX]

Table 1. Topological properties of microbial co-occurrence networks and the distribution of positive/negative correlation edges.

| Group | Point | Edge | Average degree | Modularization | Positive | Negative |
| --- | --- | --- | --- | --- | --- | --- |
| BS | 418 | 7531 | 18.017 | 0.614 | 6766 | 765 |
| CR | 440 | 8738 | 19.859 | 0.622 | 8311 | 427 |
| EN | 553 | 13304 | 24.058 | 0.554 | 12572 | 732 |
| LS | 634 | 11799 | 18.61 | 0.646 | 10937 | 862 |

Points (nodes) represent the number of microbial taxa (OTUs/ASVs) involved in the co-occurrence network, reflecting the network size. Edges denote significant correlations between taxa, indicating overall connectivity. Average degree, calculated as the average number of connections per node, serves as a core indicator of network complexity. The Modularity index measures the extent to which the network is partitioned into tightly interconnected sub-modules; higher values imply stronger clustered aggregation within the community. Additionally, positive correlation edges typically suggest mutualism, niche overlap, or synergistic metabolism among species, while negative correlation edges indicate interspecific competition, predation, or niche differentiation.
